# Behavior and Hippocampal Epac Signaling to Nicotine CPP in Mice

**DOI:** 10.1515/tnsci-2019-0041

**Published:** 2019-10-02

**Authors:** Jing Liu, Xinrong Tao, Fei Liu, Yuting Hu, Song Xue, Qi Wang, Bing Li, Rongbo Zhang

**Affiliations:** 1 Key Laboratory of Industrial dust deep reduction and occupational health and safety of Anhui Higher Education Institutes, Anhui University of Science and Technology, Huainan 232001, China; 2 Key Laboratory of Industrial Dust Purification and Occupational Health of the Ministry of Education, Anhui University of Science and Technology, Huainan 232001, China; 3 The First Affiliated Hospital of Anhui University of Science and Technology, Huainan 232001, China

**Keywords:** nicotine, Epac, Rap1, pCREB, conditioned place preference

## Abstract

Tobacco use is a major challenge to public health in the United States and across the world. Many studies have demonstrated that adult men and women differ in their responses to tobacco smoking, however neurobiological studies about the effect of smoking on males and females were limited. Exchange protein directly activated by cAMP (Epac) signaling participates in drug addictive behaviors. In this study, we examined the hippocampal Epac signaling in nicotine-induced place conditioning mice. Nicotine at 0.2 mg/kg and 0.4 mg/kg induced a conditioned place preference (CPP) in male and female mice, respectively. After CPP, male mice presented less anxiety-like behavior as demonstrated by an open-field test. The hippocampal Epac2 protein was elevated in both male and female nicotine place conditioning mice. However, Rap1 protein was elevated and CREB phosphorylation was reduced in female nicotine place conditioning mice. Our data provide direct evidence that hippocampal Epac signaling is altered in nicotine-induced CPP mice. Pharmacology manipulation Epac signaling may open a new avenue for the treatment of nicotine abuse and dependence.

## Introduction

Cigarette smoking is a leading cause of preventable death in the United States and worldwide. Approximately 15.5% of American adults are current cigarette smokers, with males (17.5%) having a slightly higher rate of use than females (13.5%) [1]. Although more men than women smoke cigarettes, some studies suggest that female cigarette smokers may be more susceptible to the negative health consequences of tobacco use. For example, females metabolize nicotine faster, more likely develop respiratory disorders, and have more difficulty with tobacco cessation than male cigarette smokers [2]. In addition, nicotine craving may be more severe in adolescent females than in adolescent males [3]. Therefore, it is important to identify the differences in behavior and biology related to nicotine exposure between males and females.

Nicotine acts on nicotinic acetylcholine receptor (nAChR) and affects multiple intracellular signaling molecules. For example, activation of α7 nAChRs increases intracellular cAMP levels via adenosine cyclase activation in hippocampal neurons [4]. Exchange protein directly activated by cAMP (Epac) is a rap1 guanine‐nucleotide exchange factor activated by cAMP. Epac modulates GTPase and alters the activities of extracellular regulated kinase (ERK), cAMP response element binding protein (CREB) and gene expression [5]. Besides modulating cardiac and smooth muscle contraction, learning and memory, cell proliferation and differentiation, apoptosis, and inflammation [6], some evidence shows that Epac signaling is altered in response to nicotine exposure. A microarray study showed that nicotine self-administration increases the medial prefrontal cortex (mPFC) Epac mRNA expression in rats [7]. Another study in humans revealed that Epac gene SNPs (rs2072115 and rs2074533) present modest association with smoking progression to nicotine dependence [8]. To date, the adaptation of Epac in response to nicotine dependence remains elusive.

The hippocampus receives extensive cholinergic innervation and expresses nAChRs at both pre- and postsynaptic compartments [9]. ACh neurotransmission modulates hippocampal-dependent function (e.g. learning and memory). Moreover, nAChR-mediated signaling is involved in the nicotine addictive behaviors [10]. Hippocampal Epac signaling regulates learning and memory [11]. *Epac* null mutation impairs long-term potentiation (LTP) and this impairment is correlated with a severe deficit in spatial learning and social interaction [12]. Therefore, we explored the interaction between nicotine use and hippocampal Epac signaling in this study in male and female mice.

Preclinical animal models are useful in examining the effect of biological responses on behaviors related to nicotine use and dependence. The conditioned place preference (CPP) task is a classical and widely used procedure to study the conditioned reward effects of addictive drugs including cocaine, morphine, ethanol and nicotine. Nicotine-induced CPP has been reported in rats [13] and mice [14]. In this study, we developed nicotine-induced CPP assay in male and female mice and explored the behaviors and hippocampal Epac signaling.

## Methods

### Animals

Adult C57BL/6 mice were purchased from the Changzhou Cavion Experimental Animal Co, Ltd. (license number SCXY (Su) 2011-0003). Mice were housed in a vivarium maintained on a standard 12 h light–dark cycle (lights on at 07:00 AM), with constant temperature and humidity (22 ℃ and 50%, respectively) and *ad libitum* access to food and water. All procedures were conducted in accordance with the guidelines as described in the National Institutes of Health’s Guide for the Care and Use of Laboratory Animals (NIH Publication No. 8023, revised 1978) and were approved by the Institutional Animal Care and Use Committee at Anhui University of Science and Technology.

### Chemicals and reagents

Nicotine was purchased from Sigma-Aldrich (N3876, St. Louis, MO). Nicotine was first dissolved in ethanol to obtain a 50 mg/mL stock solution and further diluted in sterile saline to a final concentration of 0.05 mg/ mL. α7 nAChR primary antibody (ab23832, 1:1000) came from Abcam (Cambridge, United Kingdom). Primary antibodies of Epac1 (4155S, 1:500), Epac2 (4156S, 1:1000), Rap1A/1B (4938S, 1:1000), CREB (9197S, 1:1000), and phospho-CREB (9198S, 1:1000) were purchased from Cell Signaling Technology (Danvers, MA).

### Conditioned place preference test

The construction of CPP box [15] and training [16] have been described previously. In brief, CPP apparatus (ZH-CPP, Anhui Zheng Hua biological instrument and Equipment Co., Ltd, Huaibei, China) consists of two boxes identical in size (30 × 15 × 15 cm) separated by a doorway (5 × 5 cm). One box has white walls with a gridded floor (metal bar distance 6.4 mm) and the other has dark walls with a hole floor (hole diameter 6.4 mm). The context of two boxes was calibrated to ensure most mice get roughly equal preference for either box. The mouse behavioral activity was captured with an overhead camera. Mice were habituated to recording room environment (sound-attenuated with background white noise; about 40 lux illumination) for three days. During preconditioning (day 1), the mice were allow to free access to CPP boxes for 15 minutes. During conditioning (days 2–6), nicotine-treated mice were injected with nicotine or vehicle and put in one side of the CPP box for 30 minutes. Six hours later, mice received opposite treatment (nicotine to vehicle, vehicle to nicotine) and confined to the opposite chamber for 30 minutes. Vehicle-treated mice followed the same procedure, but with two vehicle shots each day. Between trials, the bottom and walls of each box were thoroughly cleaned with 10% alcohol to eliminate interferences. During preference testing (day 7), the gate door was removed and mice were allowed to move freely into both boxes for 15 minutes. The chambers used for time recording in vehicle-treated group were matched to those in nicotine-treated group. Activity was recorded by overhead cameras.

### Open field test

One hour after CPP testing, the mouse was placed in the center of a white opaque arena (30 × 30 × 37.5 cm) and tracked via an overhead video camera interfaced with behavioral tracking software EthoVision XT 5.1 (Noldus Information Technology, The Netherlands) for 60 minutes in the same behavioral recording room. Distance traveled (the recorded movement of the mouse’s center point in cm over the duration of the trial) and immobility (the amount of time that EthoVision failed to detect any linear or angular movement of the mouse) were calculated.

### Western blotting

Right the open field test, randomly assigned mice (n=4) were deeply anesthetized by isoflurane before decapitation. The hippocampus was quickly harvested and homogenized with RIPA lysis buffer (P0013B, Beyotime, Shanghai, China) containing PMSF and phosphatase inhibitor on ice for 15 min. Hippocampal lysates were centrifuged at 10,000 ×g for 10 min at 4 ℃ to collect supernatant. The protein concentration was determined by a Bicinchoninic acid Protein Assay kit (P0009, Beyotime, Shanghai, China). Equal amount of protein (20 μg) was separated by 10% SDS-PAGE and transferred to a polyvinylidene fluoride membrane. The membrane was blocked with 5% non-fat powdered milk in TBST buffer for 1 hour before being incubated with primary antibody overnight at 4 ℃. After being washed with TBST, the membrane was incubated with goat anti-rabbit IgG H&L(HRP) (ab6721, 1:10000, Abcam) or goat anti-mouse IgG H&L(HRP) (ab6789, 1:10000, Abcam) for 40 min at room temperature. The protein blots were visualized with chemiluminescent HRP substrate (P90720, Millipore Corporation, Burlington, MA) and detected by Molecular Imager ChemiDoc^TM^ XRS^+^ analysis system (Bio-Rad Co., Hercules, CA) . Image J was used for image analysis.

### Statistical analysis

Data were expressed as mean ± SEM. All data were normal distribution. CPP data were analyzed by a paired sample Student’s *t*-test. Locomotor data and WB data were analyzed with an independent sample Student’s *t*-test (SPSS 16.0, IBM, New York). *P* < 0.05 was considered statistically significant.

## Results

### Nicotine-induced conditioned place preference

**Figure 1:**
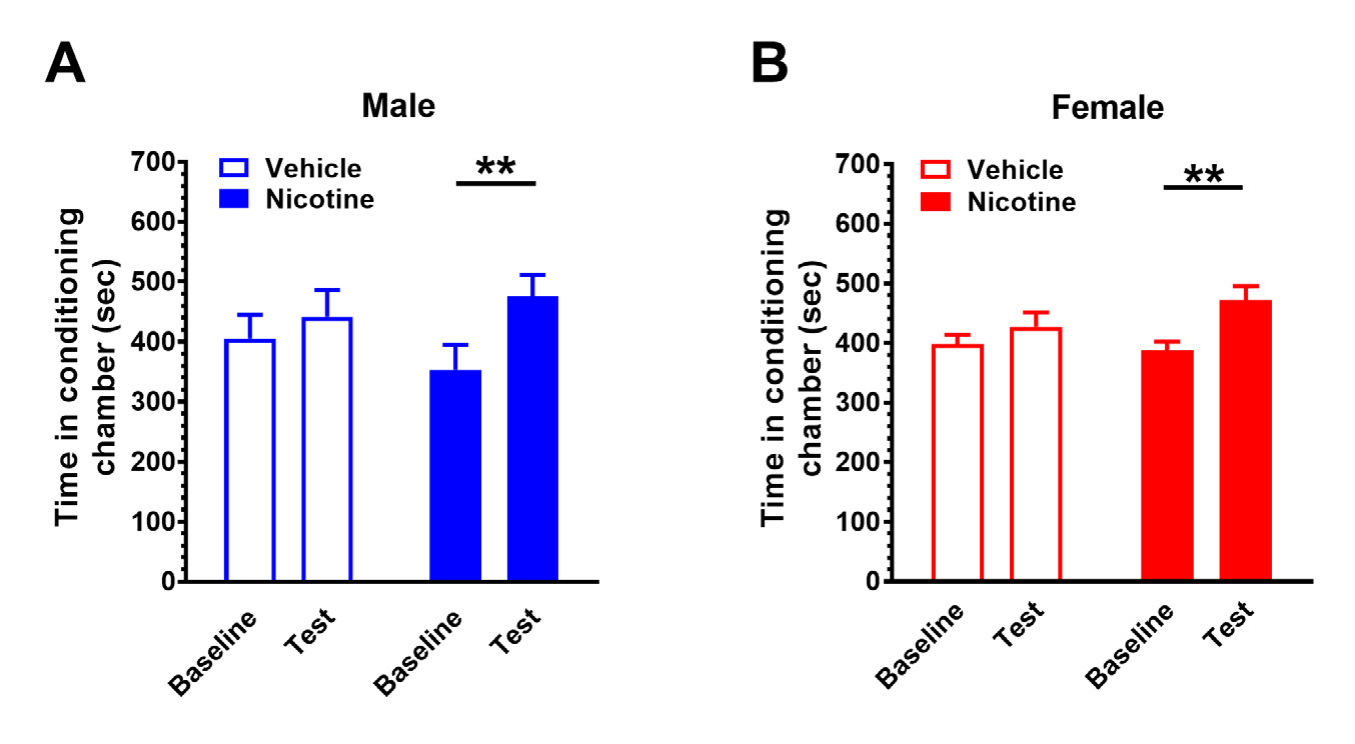
**Nicotine induces a conditioned place preference in male and female mice.** Male mice received nicotine for 5 days to induce CPP. (A) During the test session, nicotine (0.2 mg/kg, n =8)-treated male mice spent more time in nicotine associated chamber than vehicle-treated mice (n = 9). (B) Nicotine (0.4 mg/kg; n = 12)-treated female mice spent more time in nicotine associated chamber than vehicle-treated mice (n = 12). Data are expressed as mean ± SEM. **P < 0.01.

Male mice received nicotine (0.2 mg/kg_，_sc; n = 8) or vehicle (4 mL/kg, sc; n = 9) for 5 days for place preference conditioning. During the test session, nicotine place-conditioned mice spent more time in nicotine associated chamber than baseline (*t*(7) = 4.31, *P* < 0.01). Vehicle did not alter the chamber preference (*t*(8) = 0.89, *P* = 0.40; Fig. 1A) In female mice, nicotine at 0.2 mg/kg failed to develop a place preference (data not shown). In another cohort of female mice, nicotine at 0.4 mg/kg significantly increased the time spent in nicotine associated chamber compared to baseline (*t*(11) = 3.52, *P* < 0.01; n = 12). Vehicle did not alter the chamber preference (*t*(11) = 0.94, *P* = 0.37; Fig. 1B; n = 12).

### Locomotor activity in mice after nicotine place conditioning

**Figure 2:**
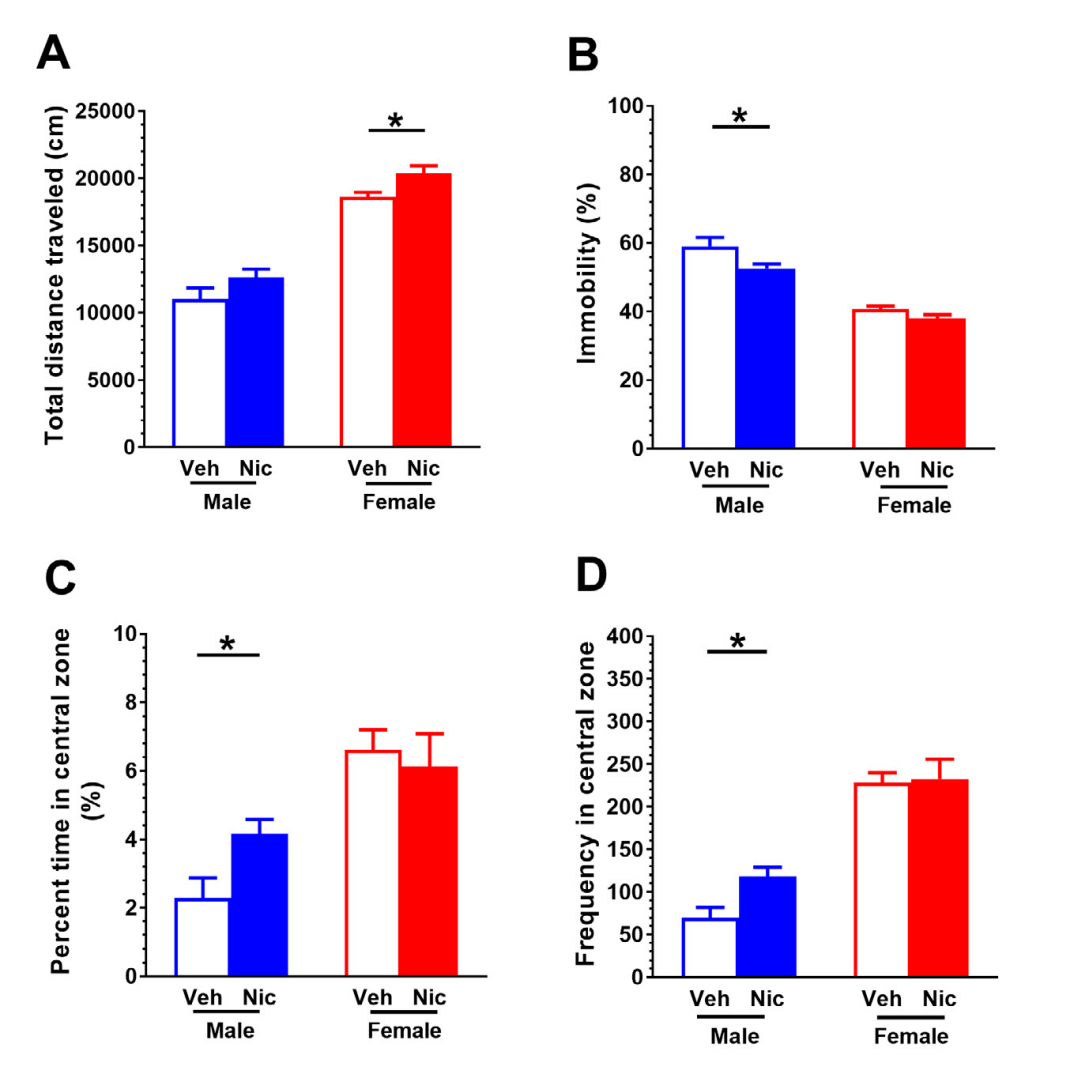
**Locomotor activity in mice after nicotine place conditioning.** The locomotor activity was recorded for 1 hour after CPP test. (A) Female nicotine place conditioned mice (n = 10) presented higher horizontal distance traveled than vehicle-treated mice (n = 12). (B) The immobility was reduced in male nicotine place conditioned mice (n = 8) as compared to vehicle-treated mice (n = 8). (C) Male mice presented an increase in central zone of the open field arena. (D) Male mice crossed the central zone more frequent than female mice. Data are expressed as mean ± SEM. *P < 0.05.

Locomotor activity was recorded in an open field arena for 1 hour after the CPP test. In male mice, there was no difference in total distance traveled (*t*(14)= -1.56, *P* = 0.14; Fig. 2A) between vehicle group (n = 8) and nicotine CPP group (n = 8), but the percentage of time in the central zone (*t*(14)= -2.592, *P* < 0.05; Fig. 2C) and time crossing the central zone (*t*(14) = -2.91, *P* < 0.05; Fig. 2D) were increased. The percentage of immobility was decreased (*t*(14)= 2.21, *P* < 0.05; Fig. 2B) in nicotine place-conditioned mice as compared to vehicle-treated mice. In female mice, nicotine CPP mice (n = 10) presented an increase in distance traveled (*t*(20) = -2.77, *P* < 0.05; Fig. 2A) as compared to vehicle-treated mice (n = 12), but there were no differences in immobility (Fig. 2B) percentage of time in the central zone (Fig. 2C) or time crossing the central zone (Fig. 2D) between these two groups (all *P* values > 0.05).

### Hippocampal α7 nAChR, PKA, Epac, Rap1, and pCREB in mice after nicotine place conditioning

**Figure 3:**
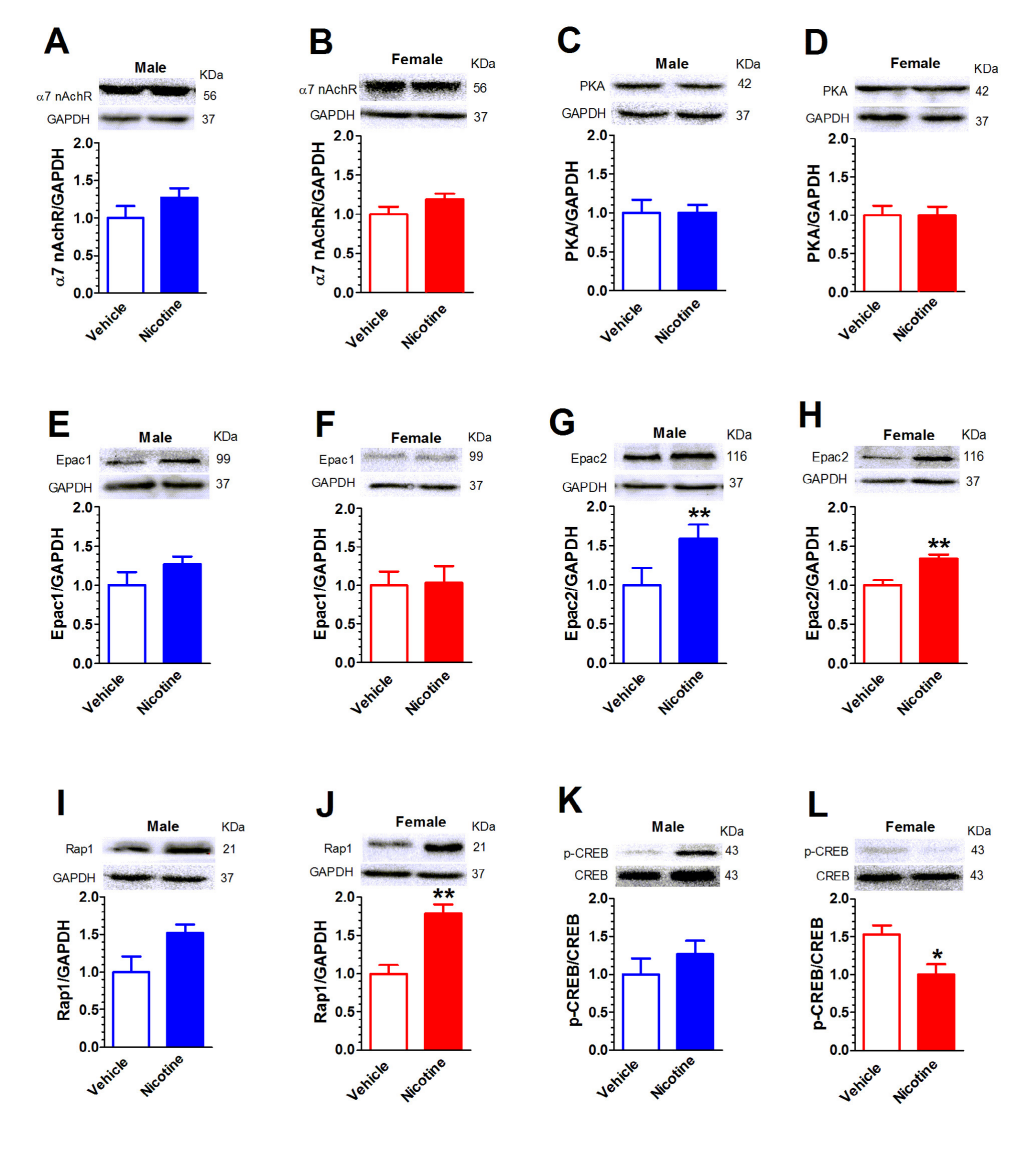
**Hippocampal α7 nAChR, PKA, Epac, Rap1, and pCREB in mice after nicotine place conditioning** There was no difference in hippocampal a7 nAChR in male (A) and female mice (B). There was no difference in PKA in male (C) and female mice (D). There was no difference in Epac1 in male (E) and female mice (F). Epac2 level was significantly increased in male (G) and female (H) mice. Rap1 expression was increase in female mice (J), but not in male mice (I). Consistently, p-CREB/CREB ratio expression was decreased in female mice (L), but not in male mice (K). Data are expressed as mean ± SEM. *P < 0.05, **P < 0.01, n = 4 in each group.

Mouse hippocampus was collected after the open-field test. Western blot assays (n = 4 in each group) revealed that hippocampal Epac2 level was significantly increased in both sexes (male: *t*(6) = -4.11, *P* < 0.01; Fig. 3G; female: (*t*(6) = -4.14, *P* < 0.01; Fig. 3H) There was no difference in a7 nAChR (male: *t*(6) = -1.31, *P* = 0.24; Fig. 3A; female: (*t*(6) = -1.59, *P* = 0.16; Fig. 3B) PKA (male: *t*(6) = -0.03, *P* = 0.98; Fig. 3C; female: (*t*(6) = -0.01, *P* = 0.99; Fig. 3D) or Epac1 of either sex (male: *t*(6) = -1.36, *P* = 0.22; Fig. 3E; female: (*t*(6) = -1.12, *P* = 0.91; Fig. 3F) Rap1 expression was significantly increased in female mice (*t*(6) = -4.72, *P* < 0.01; Fig. 3J) but not in male mice (*t*(6) = -2.29, *P* = 0.06; Fig. 3I) Consistently, the p-CREB/CREB expression ratio was decreased in female mice (*t*(6) = 2.86, *P* < 0.05; Fig. 3L) but not in male mice (*t*(6) = -0.986, *P* = 0.36; Fig. 3K)

## Discussion

The present study demonstrates that male and female mice present distinctive behavioral and biological responses to nicotine-induced place conditioning. Female mice require a higher dose of nicotine to induce a CPP. In the open field test, female nicotine place-conditioned mice presented increased overall locomotion while male nicotine place-conditioned mice spent more time in the center zone of the arena. Hippocampal Epac2 level was elevated in both male and female mice. However, an increase in Rap1 and decrease in p-CREB/CREB were only observed in female mice. The protein levels of hippocampal α7 nAChR, PKA, Epac1 and Rap1 are not different in either sex. Our data for the first time reveal the sex-specific Epac signaling in nicotine conditioning mice.

The open field test is a classic behavioral paradigm that is widely used to study animal locomotor activity and anxiety-related behaviors. We successfully developed nicotine place conditioning in both male and female mice. Prior studies have demonstrated this sexually dimorphic response in rats [17] and mice [18]. Male rats developed a significant CPP to lower doses of nicotine than females, regardless of age [19]. In line with this, we found that female mice need a higher dose of nicotine to induce a CPP. Nicotine also provokes sex-specific behavioral and biological changes [20]. For example, female mice were less sensitive to the locomotor activating effects of chronic nicotine in drinking water. Moreover, nicotine (200 μg/mL in drinking water) produced an anxiogenic-like response in females but had less effect in males [21]. Our behavioral data also reveal that female mice traveled a greater horizontal distance than male mice. However, male mice spent more time in the center zone of the open field arena after nicotine place conditioning. Although the role of sex in the mechanisms of drug action remains unclear, preclinical and clinical studies indicate that ovarian hormones, particularly estrogen, play a role in producing sex differences in drug abuse [22]. Future research is necessary to design more effective drug abuse treatment programs and resources that are sex-specific.

Epac has two isoforms: Epac1 and Epac2. Epac2 is expressed predominately in the brain, whereas Epac1 is expressed in many peripheral tissues but its expression in the brain is very low. Our data reveal that the protein level of Epac2, but not Epac1, was increased in both male and female mice after nicotine place conditioning. The role of Epac in nicotine exposure has been previously implicated. Nicotine self-administration increase the mPFC Epac mRNA expression in rats [7]. Human study found that Epac gene SNPs (rs2072115 and rs2074533) show modest association with smoking progression to nicotine dependence [8]. Our data revealed that nicotine-induced place conditioning was associated with an increase in Epac2 protein level. *Epac2*−/− mice exhibit deficits in social interaction and communication but normal locomotor activity and working memory [23]. Down-regulation of Epac2 expression in the hippocampal CA1 area impaired fear memory retrieval [11]. Along with our work, it suggests that the increase in hippocampal Epac2 expression may facilitate nicotine-induced synaptic plasticity and reinforcement learning.

Nicotine may alter hippocampus-dependent learning by changing the duration and magnitude of the activity of kinases and transcription factors involved in learning and by recruiting additional cell signaling molecules [24]. Epac downstream components include ERK and CREB [5]. Nicotine exposure and withdrawal cause distinct gene expression in PFC, amygdala, VTA and NAc in rodents. Following chronic nicotine exposure, CREB phosphorylation was reduced in the NAc and increased in the PFC, while nicotine withdrawal led to increased CREB phosphorylation in the VTA [25]. Our data revealed increased Rap1 expression and decreased CREB phosphorylation only in female mice. This difference in gene activation may attribute to the dimorphic behaviors observed and/or the higher nicotine dose used to induce CPP in female mice.

In the present study, we developed nicotine CPP mouse model and explored changes to hippocampal Epac expression and associated downstream signals. Our data directly support the association of Epac signaling with nicotine place conditioning. Further work should be done to investigate the exact molecular mechanisms of nicotine-induced CPP and Epac signaling changes due to sex difference.
